# Germinal center-dependent and -independent immune responses of tumor-infiltrating B cells in human cancers

**DOI:** 10.1038/s41423-023-01060-7

**Published:** 2023-07-07

**Authors:** Eve Playoust, Romain Remark, Eric Vivier, Pierre Milpied

**Affiliations:** 1 Aix Marseille Université, CNRS, INSERM, Centre d’Immunologie de Marseille-Luminy, Marseille, France; 2 Innate Pharma, Marseille, France

**Keywords:** antibody, tertiary lymphoid structure, plasma cell, germinal center, cancer, Tumour immunology, B cells

## Abstract

B cells play essential roles in immunity, mainly through the production of high affinity plasma cells (PCs) and memory B (Bmem) cells. The affinity maturation and differentiation of B cells rely on the integration of B-cell receptor (BCR) intrinsic and extrinsic signals provided by antigen binding and the microenvironment, respectively. In recent years, tumor infiltrating B (TIL-B) cells and PCs (TIL-PCs) have been revealed as important players in antitumor responses in human cancers, but their interplay and dynamics remain largely unknown. In lymphoid organs, B-cell responses involve both germinal center (GC)-dependent and GC-independent pathways for Bmem cell and PC production. Affinity maturation of BCR repertoires occurs in GC reactions with specific spatiotemporal dynamics of signal integration by B cells. In general, the reactivation of high-affinity Bmem cells by antigens triggers GC-independent production of large numbers of PC without BCR rediversification. Understanding B-cell dynamics in immune responses requires the integration of multiple tools and readouts such as single-cell phenotyping and RNA-seq, in situ analyses, BCR repertoire analysis, BCR specificity and affinity assays, and functional tests. Here, we review how those tools have recently been applied to study TIL-B cells and TIL-PC in different types of solid tumors. We assessed the published evidence for different models of TIL-B-cell dynamics involving GC-dependent or GC-independent local responses and the resulting production of antigen-specific PCs. Altogether, we highlight the need for more integrative B-cell immunology studies to rationally investigate TIL-B cells as a leverage for antitumor therapies.

## Introduction

Immuno-oncology research aims to deepen the understanding of the tumor microenvironment (TME) and immune cell interactions and functions to improve cancer patients’ standard of care. Tumor-infiltrating T cells have been the major focus of research and development for immunotherapies. Despite remarkable results, there is still room for improving the response rates of patients treated by T-cell targeting treatments. Tumor-infiltrating T lymphocytes (TIL-T) are not the only immune cells in tumors. B cells also frequently infiltrate solid tumors, either isolated or grouped in ectopic lymphoid formations named tertiary lymphoid structures (TLS). B-cell subsets found in tumors are named tumor-infiltrating B cells (TIL-B). In the last 5 years, numerous publications have demonstrated the favorable prognostic impact of TIL-B cells in several indications and in response to immune checkpoint inhibitors such as anti-PD(L)1 antibodies.

Despite their positive prognostic impact, the functions, interplay and dynamics of TIL-B cells in tumors remain unclear. Since B cells are fundamentally important for protective immune responses, the physiological mechanisms leading to the production of plasma cells (PCs), memory B (Bmems) cells and antibodies in secondary lymphoid organs (SLOs) have been under study for several decades. In response to activation by antigens, B cells diversify their BCR repertoires through the mechanism of affinity maturation, which occurs in germinal centers (GCs) and is a tightly regulated spatiotemporal process. Upon reinfection or reimmunization, Bmem cells can quickly differentiate into PCs in GC-independent reactions without further BCR diversification. Little is known, however, if these complex and dynamic processes actually occur within tumors.

Here, we review the essential modern knowledge about B-cell physiological dynamics in SLOs and the recent comprehension of TIL-B complex reactivities in tumors elucidated by sophisticated studies that used human samples and murine models. We highlight the different state-of-the-art methods that allow integrative TIL-B characterization in human samples and mouse models. Based on the current knowledge on B-cell responses in SLOs and on the recent TIL-B literature, we propose theoretical models of TIL-B immune responses within tumors and discuss their cellular mechanisms.

TIL-B abbreviations: For the sake of clarity, tumor infiltrating B cells are named “TIL-B”. More precisely, among TIL-B cells, tumor-infiltrating memory B cells, plasma cells, germinal centers, and naïve B cells are named TIL-Bmem, TIL-PC, TIL-GC, and TIL-Bnaive, respectively. Tertiary lymphoid structures are named TLSs. Germinal center structures in TLS-positive tumors are named GC-TLS. Conventional germinal center structures found in SLOs are named GC-SLO.

## B-cell responses in secondary lymphoid organs

B cells play essential roles in immunity, in most cases through the production of high-affinity PC and Bmem cells. We and others have recently reviewed the main mechanisms by which B cells are activated, proliferate and differentiate upon antigen exposure in SLOs [1–3]. Here, we summarize the most important concepts related to the spatiotemporal dynamics of B-cell responses after infection or immunization that are important for inferring the types of B-cell responses occurring within solid tumors.

Naïve B cells are formed in the bone marrow, where they undergo V(D)J recombination of immunoglobulin heavy and light chain genes to express a functional B-cell receptor (BCR) of the IgM isotype on their cell surface. Naïve B cells go through a selection process where their BCR functionality and tolerance toward self-antigens are tested, demonstrating an effective additional step of BCR editing. Newly released naïve B cells develop other peripheral tolerance mechanisms while circulating in the blood and traveling throughout the body to search for antigens. The encounter of a naïve B-cell with a cognate antigen is favored in specialized secondary lymphoid tissues, which are able to attract lymphocytes and concentrate antigens mainly by using antigen-presenting cells such as follicular dendritic cells (FDCs). Naïve B cells are attracted to follicles through their receptor CXCR5, which binds to the CXCL13 chemokine produced by FDCs. Naïve B cells may encounter their cognate antigen at the surface of an FDC. Upon BCR engagement with an antigen, activated B cells migrate to the T-B border of a B-cell follicle to interact and present antigens in the form of peptide-major histocompatibility complex II (pMHC-II) to recruit cognate T follicular helper (TFH) cells.

**Fig. 1:**
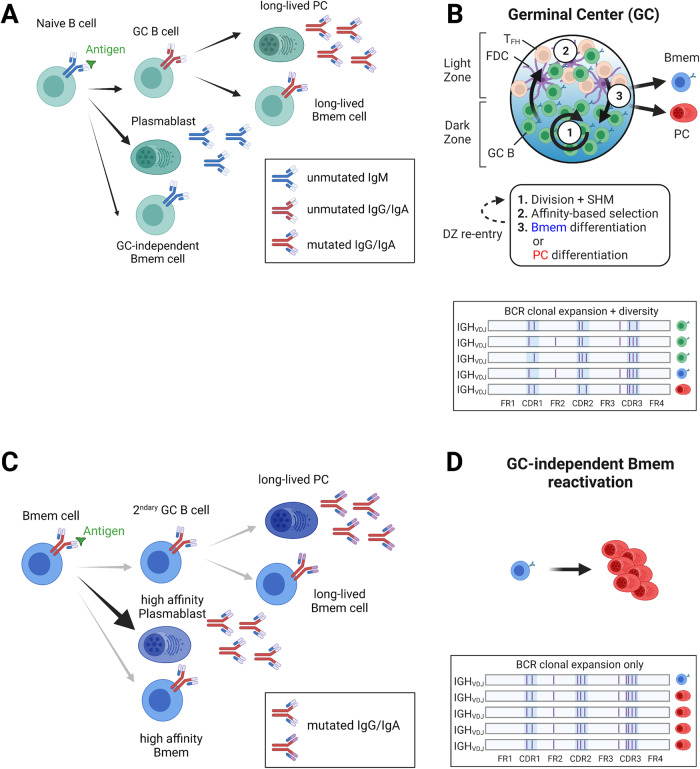
B-cell responses in secondary lymphoid organs. **A** Upon primary antigen exposure, naïve B cells may rapidly differentiate into short-lived plasmablasts producing unmutated IgM antibodies or into GC-independent Bmem cells. Naïve B cells differentiating into GC B cells will in turn generate Bmem cells and PC expressing mutated IgG or IgA. **B** Schematic representation of the GC reaction underlying the cyclic dynamics of affinity maturation. In the DZ, B cells undergo cell division and SHM. In the LZ, B cells undergo affinity-based selection through interactions with FDCs and TFH cells. After clonal selection, GC B cells can differentiate into Bmem cells or PCs or re-enter the DZ. GC-dependent clonal expansion and BCR diversification induce sequential mutations of BCR sequences and intraclonal heterogeneity. **C** Upon antigen re-exposure, Bmem cells rapidly differentiate into high-affinity plasmablasts producing mutated antibodies or into Bmem cells replenishing the Bmem pool. Bmem cells may also form secondary GCs and undergo SHM to generate a new round of long-lived PC and Bmem cells. **D** In the case of GC-independent Bmem cell reactivation, clonal expansion without further BCR diversification generates large clonotypes with no intraclonal BCR diversity. *Created with BioRender.com*

The activation of antigen-specific B cells through BCR signaling and the recruitment of cognate T-cell help may lead to three distinct fates (Fig. 1A): further activation and differentiation into germinal center (GC) B cells, differentiation into short-lived antibody-producing extrafollicular plasmablasts, or a return to quiescence and differentiation into GC-independent Bmem cells. Activated B cells proliferate extensively before differentiating; thus, a single naïve B-cell may give rise to cells that are subjected to these three distinct fates [4]. Class switch recombination (CSR) occurs in activated B cells prior to engagement in GC fate determination [5] but may also occasionally precede differentiation into extrafollicular plasmablasts or GC-independent Bmem cells. Thus, in primary immune responses, B cells expressing IgG or IgA isotypes are usually, but not exclusively, derived from naïve B cells that have been subjected to the GC differentiation process. Unlike CSR, the process of somatic hypermutation (SHM) of BCR-coding genes occurs exclusively in GC B cells during the affinity maturation process. Thus, GC-dependent PC and Bmem cells express mutated BCR genes, while GC-independent plasmablasts and Bmem cells express unmutated (germline) BCR genes.

GCs are microanatomical structures where antigen-specific B cells undergo affinity maturation (Fig. 1B). This dynamic process involves iterative cycles of cell division and SHM, followed by BCR affinity-based clonal selection of B cells. GC-dependent PC and Bmem cells may be exported after one or several cycles of affinity maturation, yielding long-lived high-affinity memory cells enabling basal protection through antibody secretion (by PC) and protection against re-exposure (by Bmem cells). Histologically, GCs are structured into a dark zone (DZ) and a light zone (LZ), which are high and low cell density regions, respectively. The LZ is populated by TFH cells and FDCs producing CXCL13, while the DZ contains a network of stromal cells that produce CXCL12. Across those regions, chemokine-driven attraction and crosstalk allow B cells to migrate bidirectionally from the DZ to the LZ and pass several checkpoints before their final GC export as differentiated effector cells. In the DZ, B cells undergo several rounds of proliferation and somatic hypermutation (SHM). Thus, DZ GC B cells express high levels of the proliferation marker Ki67 and of the AID enzyme, a cytidine deaminase responsible for SHM. The SHM process diversifies the BCR repertoire by generating mutations in variable regions of both heavy and light chain genes. Affinity for the antigen may thereby be modified. A post-SHM checkpoint determines whether a DZ B-cell’s BCR is functional before entering the LZ or dying otherwise by apoptosis. In the LZ, B cells bind and uptake antigens from LZ FDCs through their BCR and then present antigens to cognate LZ TFH cells in the form of pMHC-II complexes. High affinity BCR translates into higher cell surface densities of pMHC-II that trigger more recruitment of TFH cells that have surface CD40L expression and secrete IL21 and IL4 cytokines. LZ B cells lacking positive selection signals endure “death by neglect”. Positively selected LZ B cells may differentiate into PC or Bmem cells or return to the DZ for a new cycle of affinity maturation. In our current understanding, the fate of selected LZ B cells is partly driven by BCR affinity for antigens, with higher affinity cells favoring the PC fate and lower affinity cells favoring the Bmem fate. The BCR isotype is also a strong determinant of postselection LZ B-cell fate, with IgG^+^ and IgM^+^ favoring the PC and Bmem fates, respectively [6]. Due to those cyclic dynamics, the long-lived progeny of a GC B-cell undergoing affinity maturation express mutated variants of the original founder clone’s BCR variable genes and are thus part of the same clonotype.

Plasmablasts and PCs exit SLOs through the lymphatics and are transiently found in blood but establish long-term residency in specific regions, mostly in the bone marrow, where they are retained through the CXCR4-CXCL12 signaling axis. After being exported from GC reactions, Bmem circulate through the blood and migrate to other SLOs, where they will be ready to detect antigens upon secondary exposure in the future. Upon reactivation, high-affinity isotype-switched Bmem cells may undergo the same three fates as naïve B cells (Fig. 1C). However, their fate mainly results in rapid proliferation and differentiation into GC-independent extrafollicular plasmablasts that produce large amounts of isotype-switched high-affinity antibodies with strong protective effects. Some reactivated Bmem cells re-enter or reform secondary GCs, where they compete with de novo activated naïve B cells for further affinity maturation and differentiation [7]. The GC-independent differentiation of Bmem generates large numbers of clonally related plasmablasts expressing BCR sequences with the same pattern of somatic mutations as their Bmem ancestor (Fig. 1D). Notably, Bmem cell reactivation not only takes place in SLOs but may also occur directly at the site of infection in peripheral tissues (e.g., in pulmonary viral infections), resulting in local clusters of tissue-resident antigen-specific Bmem cells and PCs [8, 9].

Altogether, B-cell responses in SLOs involve GC-dependent and GC-independent pathways yielding short-lived and long-lived Bmem and PC progeny. Expression of a somatically mutated BCR implies affinity maturation through a GC reaction at some stage during the clonal history of the Bmem cell or PC. The pattern of intraclonal BCR variable sequence diversity discriminates recent GC-dependent versus GC-independent activation and differentiation.

### TIL-B prognostic and predictive value

In recent years, TIL-B cells have been revealed as important players in antitumor responses in human cancers. Several reviews have extensively described their positive prognostic value in several conditions [10–15].

A recent clinical trial studying the impact of pembrolizumab (anti-PD1) combined with low-dose cyclophosphamide in patients with advanced soft tissue sarcoma (STS) demonstrated the clinical value of assessing TIL-B infiltration for patient prognosis [16]. The 30 patients who had been enrolled based on the detection of TLS in their tumors showed a significant survival improvement (6-month non-progressive rate (NPR) of 40%) compared to other patients in the clinical trial (6-month NPR of 4.9%). This clinical trial followed the publication of Petitprez et al. [17] showing an immune cell gene expression-based classification of STS, a highly heterogeneous group of cancers. The immune cell group within the tumors was enriched in TIL-B cells, T cells, and follicular dendritic cells and contained TLS. TIL-B infiltration was the strongest prognostic factor independent of the presence of CD8^+^ T cells, suggesting that TIL-B cells could play a role in the response to immune checkpoint inhibitor therapies. A recent large-scale retrospective analysis of three independent cohorts also revealed that the presence of TLS containing germinal centers (GC-TLSs), named mature TLS in this study, was predictive of checkpoint inhibitor efficacy independently of PD-L1 expression or CD8^+^ T-cell densities [18].

Beyond the prognostic importance of GC-TLS, a transcriptional analysis in large cohorts of lung cancer patients revealed that the dominant signature associated with improved overall survival upon anti-PD-L1 treatment was a TIL-PC signature. In this study, the presence of TIL-PCs was also associated with the presence of lymphoid aggregates and TLS [19].

TLS and TIL-Bs are now in the spotlight of immuno-oncology as potential key actors for patient survival and response to ICIs. Nonetheless, their interplay and dynamics within tumors remain largely unknown compared to our deep understanding of B-cell dynamics in SLOs in the context of infectious diseases or vaccination.

### Methods for studying B-cell responses in tumors

**Fig. 2:**
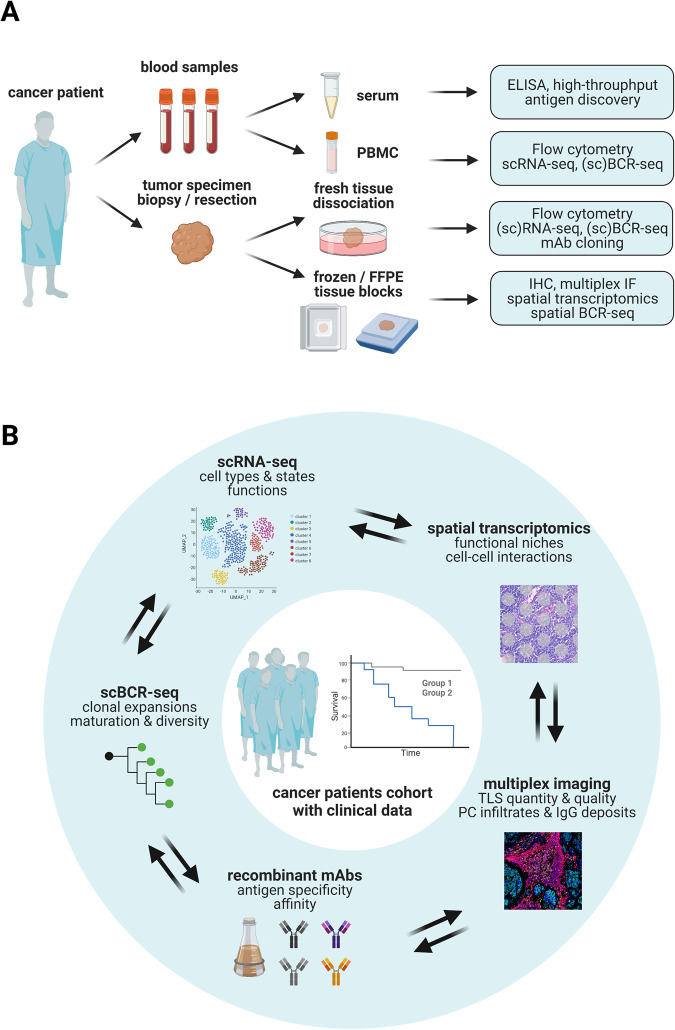
Integrative methods for studying B-cell responses in tumors. **A** Depending on the material that is available, different immunology, genomics, and imaging methods may be used to study B-cell responses in cancer patients and their tumors. Blood samples may be used to study antibodies in the serum, B-cell subsets and BCR repertoire among peripheral blood mononuclear cells (PBMCs). Tumor samples may be used fresh for studying the immune cell composition by flow cytometry and single-cell RNA-seq and as a source of live B cells and PCs for single-cell cloning of BCR and production as recombinant monoclonal antibodies. Frozen or formalin-fixed paraffin-embedded (FFPE) tissues may be used for histology, imaging, spatial transcriptomics and BCR sequencing. **B** A deeper understanding of TIL-B responses and their importance will come from integrating several B-cell-focused analyses on tumor and blood samples from well-annotated clinical sample cohorts. Created with BioRender.com

The same methods that are used for studying B-cell responses in SLOs are used for studying B-cell responses in tumors (Fig. 2A). Although some mouse models of tumor development may recapitulate the B-cell infiltration levels that are observed in human tumors [20], most of the published knowledge on TIL-B-cell diversity comes from analyzing human samples. Blood is the most accessible sample type in human patients but offers only a limited time period to observe circulating B-cell subsets and antibodies. Tumor samples may be collected after resection surgery or biopsy. Resection specimens are larger and may be used fresh for analysis by flow cytometry, single-cell RNA-seq, B-cell cloning or functional assays. In flow cytometry staining, naïve B cells (CD19^+^CD20^+^IgD^+^CD27^−^CD38^−^), Bmem (CD19^+^CD20^+^IgD^−^CD27^+^CD38^−^), GC (CD19^+^CD20^+^IgD^−^CD10^+^CD38^+^), plasmablasts (CD19^+^CD20^−^IgD^−^CD27^+^CD38^hi^) and PC (CD19^lo^CD20^-^IgD^−^CD27^+^CD38^hi^CD138^+^) may be discriminated based on their expression of specific surface markers [21, 22]. Among GC B cells, LZ and DZ cells can be further discriminated based on their expression of CXCR4 (DZ) and CD83 (LZ) [22, 23].

In single-cell RNA-seq, non-PC B-lineage cells are identified by the expression of several B-cell-specific transcripts (e.g., *MS4A1*, *CD19*, *CD79A*, *CD79B*), and PC lineage cells are identified by the high expression of *IGH*, *IGK* and *IGL* transcripts (e.g., *IGHM*, *IGHG1*, *IGHA1*, *IGKC*) and other genes related to antibody production (e.g., *XBP1*, *DERL3*) [24–26]. PCs express two orders of magnitude more *IG* transcripts than non-PCs and are clustered based on heavy chain and light chain isotypes when IG genes are retained in the highly variable genes computed before dimensionality reduction and clustering; this process leads to different clusters of PC that share the vast majority of their marker genes but differ based on their isotype [25, 27]. Collapsing all *IGH* and all *IGK* or *IGL* transcripts into unique pseudogenes enables us to focus the analysis on gene expression differences that are independent to the BCR isotype class [24]. Among antibody-producing cells, plasmablasts (PBs) express cell cycle-related transcripts (e.g., *MKI67* and *STMN1*). Among non-PC cells, the expression of characteristic marker genes discriminates naïve B cells (*IGHD*, *FCER2*, *TCL1A*, *IL4R*), Bmem cells (*BANK1*, *BACH2*, *SELL*, *CD27*), and occasionally GC B cells (*BCL6*, *RGS13*, *AICDA*). Finer subsets and activated states represented by minor populations of cells can be discriminated when large numbers of cells are analyzed [28, 29]. Single-cell RNA-seq may be coupled with single-cell BCR sequencing when the full-length cDNA or the 5′-end of cDNA is used as a target for cell barcoding and sequencing, thereby connecting the BCR sequence to the transcriptome and enabling tracking of isotype diversity, somatic hypermutation patterns and clonal lineages of B cells in SLOs or tumors [27, 29].

Fresh resection specimens may also be divided into different samples for archiving as fresh-frozen and FFPE blocks. Biopsy specimens are much smaller and usually only conserved as FFPE blocks. Recent imaging and genomics techniques enable the collection of large amounts of descriptive data on small amounts of tissue materials. Compared with single-marker immunohistochemistry (IHC), multiplex immunofluorescence (IF) assays allow the analysis of several markers on a single tissue slide, thereby saving tissue material and enabling the characterization of complex cellular phenotypes in situ. Multiplex IF has already been widely used for the characterization and quantification of TLS in large cohorts of patients [17, 18, 30, 31]. TLS are usually identified by CD20 staining, and the maturation state of TLS is inferred from the presence of CD23^+^ FDCs and/or Ki67^+^ proliferating B cells [32]. Bulk RNA sequencing of archived tissue, when combined with reference-based deconvolution of cellular phenotypes by bioinformatic algorithms, also enables the semiquantitative analysis of immune infiltrates [33, 34]. If the RNA quality is high, such datasets may also be used for adaptive immune receptor repertoire (BCR and TCR) analysis [35, 36]. Spatial transcriptomics methods enable the mapping of gene expression for hundreds to thousands of genes directly in tissue slides [37]. The most spatially resolved approaches yield subcellular resolution but can currently only detect up to 1000 preselected genes through probe hybridization. The less resolved spot-based approaches capture the full transcriptome from small areas of tissue (55-µm diameter spots for the popular Visium slides), requiring reference-based bioinformatic deconvolution to quantify the composition in cell types and states of the tissue above each spot [38] but yielding spatially barcoded full-length cDNA that can be used for spatial analysis of BCR and TCR sequences [39, 40]. In the near future, a deeper understanding of TIL-B responses and their importance may be generated by integrating several B-cell-focused analyses on tumor and blood samples from well-annotated clinical sample cohorts (Fig. 2B).

### TIL-B subsets in human solid tumors

Single-cell RNA-seq or flow cytometry studies of fresh tumor samples have detailed TIL-B subset proportions and frequencies in several scenarios.

Germain et al. [21] have identified abundant TIL-Bmem levels (70%), intermediate levels of TIL-PC (15%) and low TIL-Bnaive (less than 10%) and TIL-GC levels (less than 5%) among CD19^+^ cells in fresh tumor samples from NSCLC patients by flow cytometry. Single-cell studies performed by Lambrechts et al. [25] comparing nontumoral to tumoral lung samples revealed a strong enrichment of TIL-B cells inside tumors. Clustering TIL-B cells revealed the presence of follicular B cells expressing high levels of *MS4A1*, *CXCR4*, and *HLA-DR*, alongside IgG^+^ and IgA^+^ TIL-PCs, the latter being named MALT-B cells by the authors. Laughney et al. [41] also reported an enrichment of TIL-B cells in primary lung adenocarcinoma (LUAD) samples compared to metastatic (adrenal, bone or brain metastasis) or nontumoral samples. Kim et al. [42] studied several tissues, including normal lung, tumoral lung, normal lymph node, and metastatic lymph node, from early to advanced LUAD patients. They identified several TIL-B clusters that were preferentially enriched in specific tissues. TIL-GC transcripts were very scarce in tumoral samples (less than 1%) but were increased in metastatic LN compared to normal lung tissues. MALT B cells (20%) and TIL-PCs (5%) were mostly represented in tumor samples and metastatic brain samples. Metastatic LNs had less TIL-PC than normal LNs. Qian et al. [26] profiled single cells of tumors from 36 patients with lung, colorectal, ovary or breast cancer. Among 15,247 TIL-B cells, they identified eight main clusters divided into follicular B cells and TIL-PCs. Among follicular B cells, they identified abundant TIL-Bmems but rare TIL-Bnaive and IgM^+^ TIL-Bmems. IgG^+^ and IgA^+^ TIL-PCs were equally represented in lung and ovarian tumors (nearly 50% of all TIL-B) and more abundant in CRC samples (more than 50%) because of a higher proportion of IgA^+^ TIL-PCs. In a head and neck cancer sample, Wieland et al. [43] identified 79% of activated B cells, 16% of TIL-PCs, and 4% of TIL-GCs by scRNA-seq. Similarly, Ruffin et al. [44] identified by high-dimensional flow cytometry 20% of TIL-Bnaive, 40% of TIL-Bmem, and 10% of TIL-PC among the CD19^+^ B cells in a head and neck cancer sample. Interestingly, the frequency of mature TLS containing TIL-GC cells is higher in human papillomavirus (HPV)-positive head and neck tumors [44], and TIL-PCs infiltrating HPV-positive head and neck tumors are frequently reactive to HPV antigens [43], suggesting that tumors developing at barrier tissues in contact with foreign antigens may be most likely to develop TIL-GC structures.

In inflammatory conditions, subsets of immunoregulatory B cells, mostly characterized by their capacity to produce IL-10 and/or IL-35 and collectively named Breg cells, have been described as critical negative regulators of immune responses [45, 46]. Breg cells in cancer have been studied in various mouse models and human samples (as reviewed here [47]); Breg cells have notably been associated with the progression and immune escape of pancreatic cancer [48, 49]. In single-cell RNA-seq studies of human TIL-B cells, rare B-lineage cells expressing the *IL10* transcript to detectable levels were not enriched in a particular cluster (subset) in unsupervised analyses [27, 50], which is in line with the diverse phenotypes of IL-10-producing B cells in ex vivo activation studies [51].

In summary, flow cytometry and scRNA-seq analyses have revealed that TIL-B subsets are enriched in human solid tumors compared to their nontumoral tissue counterparts, with the most abundant subsets being TIL-PCs and TIL-Bmems cells. TIL-GC was detected in most studies but was often the rarest TIL-B subset. Breg cells may play important regulatory functions but do not correspond to a phenotypically distinguishable subset of TIL-B cells.

### TIL-B spatial organization: TLS and GC-TLS structures

TIL-B subsets are frequently organized using TLS. TLS organization and the mechanisms driving their formation in tumors have been extensively reviewed elsewhere [15, 52]. TLS display various organization and maturation degrees that have been well described by Silina et al. [32] using imaging analyses of lung tumor samples. They defined early TLS (E-TLS), formed by a mixture of T (CD3^+^) and B (CD20^+^) cell aggregates, primary-follicle-like TLS (PFL-TLS) containing FDC (CD21^+^) networks to support TLS organization, and secondary-follicle-like TLS (SFL-TLS) containing GC-like structures (GC-TLSs) with CD23^+^ TIL-GCs. GC-TLS are also identified by the expression of BCL6 [53], a master transcriptional regulator of TFH and GC B cells, and the proliferation marker Ki67 [21]. From a structural perspective, GC-TLS are formed by the aggregation of TIL-GC cells into imperfect circular clusters.

**Fig. 3:**
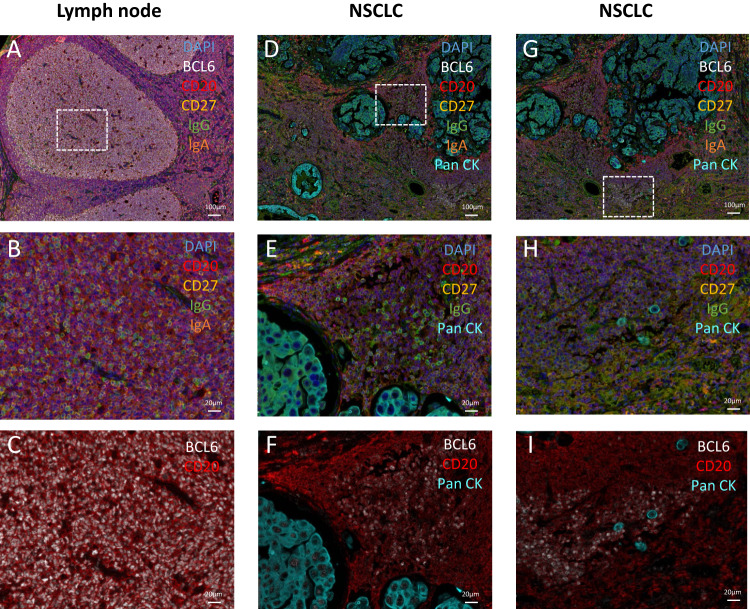
GC-TLS displays structural differences from conventional GC-SLO. Multiplex IF staining of a TIL-B infiltrated non-small cell lung cancer (NSCLC) tumor sample compared with a reactional lymph node to assess the presence of B-cell subsets and GC organization. Staining was performed with the Leica Bond Rx. Our multiplex panel was designed for TIL-B identification: mouse anti-CD20 (clone L26, DAKO), rabbit anti-BCL6 (EPR11410-43, Abcam), rabbit anti-IgA (EPR5367-76, Abcam), rabbit anti-CD27 (EPR8569, Abcam), rabbit anti-IgG (EPR4421, Abcam), and anti-pancytokeratin (AE1/AE3/PCK26, Roche). We used the Akoya Bioscience Opal 6-Plex Detection Kit using TSA opal fluorophores (Opal 480, Opal 520, Opal 570, Opal 620 and Opal 690). Slides were counterstained with spectral DAPI (Akoya Bioscience), cover-slipped, and scanned using the PerkinElmer Vectra Polaris System. **A**–**C** Representative images of GC-SLO. **D**–**I** Representative images of GC-TLS. Low magnification (scale bar = 100 µm) images with all markers are shown in (**A**, **D**, **G**). White squares indicate areas selected for higher magnification views. High magnification (scale bar = 20 µm) images with only the indicated markers are shown in (**B**, **E**, **H**, **C**, **F**, **I**)

Using multiplex IF imaging (Fig. 3), we compared a healthy human reactive LN with a human NSCLC sample to assess the similarities between GC-TLS and conventional GC-SLO. We identified the presence of Bnaive cells (DAPI^+^ CD20^+^ CD27^-^), Bmem cells (DAPI^+^ CD20^+^ CD27^+^) and IgA^+^ or IgG^+^ PC (DAPI^+^ CD20^-^ IgA^+^ or IgG^+^) in both samples. In rare GC-TLS, we identified germinal center B cells (DAPI^+^ CD20^+^ BCL6^+^), but GC-TLS sizes were much smaller than those of GC-SLOs. Additionally, BCL6^+^ cell density was lower in GC-TLS. Ruffin et al. [44] studied GC-TLS of HPV-positive head and neck tumor samples in comparison with conventional GC-SLO from tonsils. They observed strong Sema4a expression in both GC structures and documented higher Sema4a expression in LZ B cells by flow cytometry. DZ-LZ organization was not obvious in GC-TLS stained for Sema4a by IHC. In primary and metastatic melanoma samples, Werner et al. [53] identified mature TLS containing GC but failed to identify DZ-LZ polarization of GC based on Ki67 (DZ marker) and CD21 (LZ FDC marker).

Thus, tumor TLS have different degrees of maturity, the most mature TLS presenting GC-like structures and TLS-GC cells with true phenotypic and transcriptional GC markers. However, GC-TLS structures are usually small and seem to lack the DZ-LZ polarization that is characteristic of GC-SLO and required for the cyclic process of affinity maturation.

### TIL-B interactions in response to ICI treatments

Tumor infiltration with B cells is associated with a favorable response to immune checkpoint inhibitor (ICI) anti-PD(L)1 treatment that is designed to reinvigorate exhausted T cells (mostly CD8^+^ cytotoxic T cells) in tumor microenvironments. B cells themselves may express PD-1 in certain activation or inflammatory conditions [54–56], and PD-1^hi^ B cells have been described as playing an immunoregulatory role in hepatomas [57]. However, most studies reported thus far suggest that the favorable prognostic role of B cells in response to ICI treatment may be due to indirect mechanisms through B-T-cell collaboration (see below).

Cabrita et al. [30] showed in melanoma that the co-occurrence of CD20^+^ TIL-B and CD8^+^ T cells was associated with improved patient survival. They found an increase in TCF7^+^ naïve or memory T cells in TIL-B rich tumors. In contrast, T cells in tumors deprived of TLS displayed a dysfunctional phenotype. Patil et al. [19] revealed that TIL-PC infiltrate was positively correlated with the presence of T cells organized in TLS or lymphoid aggregates in NSCLC tumors treated with anti-PD-L1. Vanhersecke et al. [18] suggested a role for CD8^+^ T cells and TIL-B collaboration in response to anti-PD-1 treatment, even if the main prognostic impact was conferred by mature TLS (GC-TLS) in their study. Indeed, patients with poor CD8^+^ T-cell infiltration in their tumors had poorer outcomes regardless of TLS status, suggesting that CD8^+^ T-cell presence was necessary but not sufficient to generate a robust antitumor response. In NSCLC, Thommen et al. [58] highlighted the ability of exhausted PD-1^hi^ CD8^+^ T cells to recruit B cells via CXCL13 secretion upon anti-PD-1 treatment. The presence of those PD-1^hi^CD8^+^ T cells was predictive of patient response to anti-PD-1 immunotherapy.

These descriptive studies in human tumor samples shed light on potential B-T-cell collaboration in antitumor responses upon ICI treatment. Studies in murine models have explored the possible mechanisms that may be involved. Rodriguez et al. [59] used a murine melanoma model in which ICI treatment induced larger and more organized TLS. CD8^+^ T cells were involved in the initial aggregation and reticular network formation of cancer-associated fibroblasts (CAFs), while B cells recruited via CXCL13 drove CAF proliferation and the expansion of TLS through lymphotoxin beta/lymphotoxin beta receptor (LTBR) crosstalk. In a murine breast cancer model with high mutational burden, Hollern et al. [60] showed that B-TFH cell interactions were required to mediate ICI treatment response, which in turn involved PC secretion of antibodies. Depletion of T regulatory (TREG) cells also improved antitumor responses in this model.

Therefore, functional collaborations between TIL-B and CD8^+^ T cells are likely involved in the response to ICI treatment.

### Antigen-restricted B-TFH interactions

Regardless of ICI treatments, several studies based on murine models have shed light on potential T-B cell interactions providing antitumor responses using systemic T-B-cell depletion and adoptive transfer manipulation. Cui et al. [61] studied B-TFH cell interactions in a murine model of lung adenocarcinoma (LUAD) in which tumor cells express neo-antigens recognized by B and T cells or T cells only. They showed that tumor antigen recognition by B and T cells was necessary for naïve T-cell differentiation into TFH cells producing IL-21. In turn, IL-21 potentiated CD8^+^ T-cell cytotoxicity via granzyme B upregulation, enhancing antitumor responses. In a mouse model of breast cancer treated by chemotherapy, B-cell-specific deletion revealed that ICOSL expression in B cells is important for B-T-cell interactions in TLS [62]. ICOSL expression in B cells was dependent on the complement receptor CR2 and antigen recognition via BCR, triggered by immunogenic cell death. The presence of ICOSL^+^ B cells led to a higher Teffector/TREG ratio and improved antitumor immunity.

These studies highlight B-TFH interactions that are beneficial to the antitumor immune response. It is still unclear whether beneficial B-TFH interactions occur primarily within tumor TLS or in tumor-draining SLOs.

### Potential GC-independent antigen-specific TIL-B responses

There is currently little evidence from studies on human tumors that GC-TLS reactions are productive. The frequent high density of TIL-PCs contrasts with the rare occurrence of low numbers of small GC-TLSs and questions the ability of GC-TLSs to generate such a high number of TIL-PCs. Could the majority of TIL-PCs be generated in GC-independent responses? Or could they be recruited from SLOs in permissive tumor microenvironments?

In a spatial transcriptomic analysis of human renal cell carcinoma (RCC) samples, Meylan et al. [63] showed that TIL-PCs were clustered along CXCL12^+^ fibroblast reticular tracks. The CXCR4-CXCL12 axis is well known for regulating long-lived PCs in the bone marrow. CXCL12^+^ stromal cells in the medullary area of SLOs are also known to provide signals such as BAFF or APRIL to promote B-cell survival [1–3]. Thus, it is likely that CXCL12^+^ stromal cells in tumors function as a niche to host TIL-PCs that are either generated locally or recruited from the periphery. Meylan et al. [63] favor the former hypothesis because they observed that those CXCL12^+^ tracks extend from TLS areas within the tumor. They also associated the presence of TLS and TIL-PCs with IgG deposits on tumor cells, suggesting that TIL-PCs secrete tumor antigen-specific antibodies. In ovarian cancer, Biswas et al. [64] showed that secreted IgA bound the polymeric immunoglobulin receptor (pIgR) on ovarian tumor cells, leading to IgA transcytosis across them. Transcytosis induced transcriptional changes within tumor cells and sensitized them to T-cell killing. In that study, in situ IgA production also led to tumor cell death after activation of antibody-dependent cell phagocytosis by myeloid cells.

### Inferring TIL-B origin from the BCR repertoire

As shown in Fig. 1, the patterns of BCR sequence mutations within clonally related B cells are informative on the type of B-cell responses they have undergone. Can we use BCR-seq analyses of TIL-B cells to assess the relative contributions of GC-dependent and GC-independent responses?

In conventional GC-SLO reactions generated by primary immunization, the SHM load in Bmem and GC B cells rarely exceeds ten nucleotide mutations in the heavy and light chain variable genes [65]. Higher numbers of somatic mutations in BCR repertoires reflect a longer history of affinity maturation, either in chronically activated GC, through several rounds of GC re-entry, or through GC-independent activation of highly mutated Bmem cells. In human ovarian carcinoma tumors, Mazor et al. [66] studied the clonal diversification of TIL-PCs through single-cell BCR-seq. Nearly 50% of TIL-PC clonotypes were expanded. The average SHM load in IGHV genes was approximately 20, and lineage tree reconstructions revealed that most large clonotypes had diversified extensively from their unmutated common ancestor. Some clonal trees exhibited progressive SHM accumulation and clonotype diversification, suggesting that some TIL-PCs were regularly exported from ongoing memory GC reactions. Other clonal trees showed large clonal expansions without diversification, suggesting GC-independent Bmem reactivation and differentiation. Wieland et al. [43] analyzed TIL-PC clonal expansions in HPV-positive head and neck carcinoma samples. HPV antigen-specific TIL-PCs had high levels of SHM (on average 28 mutations in IGHV sequence). Meylan et al. [63] also measured average IGHV SHM loads between 15 and 18 in TIL-PC sequences of renal cell carcinoma samples.

In all cases, the SHM levels reported are consistent with most TIL-PCs being derived from Bmem cell reactivation and differentiation into PCs and seem to imply a long history of affinity maturation rather than recent output from a primary GC. Bmem cells are able to re-enter ongoing GC under certain conditions [67] but more frequently directly differentiate into plasmablasts upon antigen activation [7]. In healthy humans, at steady state, circulating plasmablasts may be derived from the same Bmem cells over several years [68], suggesting that GC-independent Bmem reactivation is frequent even in the absence of acute antigenic stimuli. Such Bmem reactivation likely occurs in SLOs but may also take place within tumor TLSs. Lung-resident Bmem cells have been shown to be a potent source of local antigen-specific antibody-producing PC in airway viral infections [8, 9], which suggests that similar mechanisms may be occurring with tumor-resident Bmem cells.

### TIL-B antigen specificity

What antigens are recognized by TIL-B cells? Many tumor-specific antigens were historically identified by screening serum antibodies against tumor cell-derived cDNA expression libraries with the serological identification of antigens by the recombinant expression cloning (SEREX) method [69]. Serum reactivities against known tumor antigens such as P53, MAGEA4, SOX2 and NY-ESO have been extensively studied in patient sera and may also be used as biomarkers of cancer [70].

Recent reports in mice and human tumor samples [60, 63, 64, 66, 71] have demonstrated the existence of immunoglobulin deposits on tumor cells in situ. In Biswas et al. [64], OVCAR3 ovarian carcinoma cells were coated with tumor-derived IgA. This binding was abrogated when IgA was replaced with IgG or when pIgR was deleted from OVCAR3 cells. Additionally, in ovarian carcinoma, Mazor et al. [66] detected IgG-coated tumor cells in a significant fraction of samples (61%) compared to healthy epithelium of the fallopian tube or the ovary. They also reported the presence of numerous IgG^+^ TIL-PCs in the tumor samples. This finding is consistent with the observations from Meylan et al. [63] investigating renal cell carcinoma, where tumor cells are frequently coated by IgG, especially in samples with dense TIL-B and TIL-PC infiltrates. In a high mutation burden mouse breast cancer model, Hollern et al. [60] also identified IgG binding of tumor cells upon anti-PD-1 and anti-CTLA-4 antibody treatment. In another mouse breast cancer model studying the impact of IgG production on metastatic spreading of tumor cells, Gu et al. [71] reported pathologic IgG binding to the membrane of 4T1 tumor cells, a mechanism not observed in healthy mice upon 4T1 cell treatment with IgG.

Thus, TIL-PC-derived antibodies may target antigens expressed by tumor cells. However, it is unclear what proportion of TIL-B and TIL-PC express tumor-specific antibodies and what antigens are targeted.

Wieland et al. [43] identified high frequencies of HPV protein-specific TIL-PC in HPV-positive head and neck carcinoma samples. Those TIL-PCs targeted the E6 and E7 oncogenes and the regulatory protein E2. On average, 1–2% of IgG^+^ TIL-PCs targeted one of those HPV antigens in tumors and metastatic lymph nodes, with frequencies reaching 10% in some tumors. TIL-PC viral antigen specificity was correlated with serum titers against viral antigens in HPV-positive patients. Overacre-Delgoffe et al. [72] studied the introduction of *Helicobacter hepaticus* (*Hhep*), an immunogenic intestinal bacterium, in a mouse model of colorectal cancer. *Hhep* colonization favored the antitumor immune response by inducing *Hhep*-specific TFH cells and mature TLS, demonstrating that TLS-GC-dependent B-cell responses targeting nontumor foreign antigens could also benefit the antitumor immune response.

HSPA4 protein was identified by Gu et al. [71] as a tumor glycosylated antigen implicated in lymph node metastases in a mouse model of breast cancer. Tumor-bearing mice had higher serum IgG levels, which could bind glycosylated HSPA4 proteins. HSPA4 binding triggered the CXCR4-CXCL12 axis to promote lymph node infiltration, a prerequisite for tumor metastasis development in lymph nodes. Here, autoreactive HSPA4-specific TIL-B seemed to be implicated in the establishment of a premetastatic niche. Patient sera also showed reactivity against HSPA4, which was correlated with poor prognosis and was also correlated with HSPA4 expression in tumors. In malignant mesothelioma, the mesothelin protein (MSLN) is highly expressed. Liu et al. [73] reported a strong antitumor response following treatment with LMB-100, a recombinant immunotoxin composed of an MSLN-targeting Fab linked to a toxin, in an immunocompetent mesothelioma mouse model. LMB-100 triggered antitumor immunity depending on B and T cells that were not restricted to the MSLN antigen, suggesting that an immune response generated toward one antigen could lead to durable antitumor responses against other tumor cell antigens.

In ovarian cancer, Biswas et al. [64] used human proteome arrays to test the specificity of IgA antibodies cloned from TIL-PCs. They identified two extracellular antigens, BDNF and TSPAN7, a secreted molecule associated with poor prognosis in ovarian carcinoma and a tetraspanin overexpressed in ovarian carcinoma, respectively. Importantly, anti-BDNF or anti-TSPAN7 IgA treatment slowed tumor growth in vivo through mechanisms involving antibody-dependent cell cytotoxicity and phagocytosis. Mazor et al. [66] produced monoclonal antibodies cloned from ovarian cancer TIL-PCs. They discovered that a majority of antibodies were reactive against primary tumors and tumor ascite-derived cell cultures. They tested the reactivity of antibodies against matrix metalloproteinase (MMP) antigens because ovarian cancers are known to be involved in tissue remodeling and express high levels of those enzymes. For a majority of antibodies, they found strong reactivity against MMP14, with some reactivity against other MMPs. The high abundance of MMPs in the tumor microenvironment may be sufficient to cause a break in tolerance and promote the generation of autoreactive antibodies recognizing both tumor cells and healthy tissues. By reverting the anti-MMP14 recombinant antibodies to their unmutated common ancestor (germline) sequences, they identified two classes of antitumor antigen antibodies; class I antibodies lost their binding capacities toward MMP14 once reverted to their germline sequence, while class II antibodies bound equally well as germline. This suggests that tumor- and self-reactivity of TIL-PCs may be acquired through affinity maturation in GC (class I) or may be preexisting (class II).

Despite interesting recent findings, a substantial amount of research is still needed to understand the antigen specificities of TIL-B subsets. To date, there is no way to computationally infer the epitope structure or sequence from the BCR sequence, although that challenge may be solvable by new technologies [74]. Pairing broad antigen-specificity assays with single-cell BCR sequencing [75] on a massive scale will be important to generate sufficient data for training artificial intelligence models that can be designed to resolve that issue.

## Conclusions and perspectives

Recent evidence has put TIL-B in the spotlight of cancer immunotherapy research. Their organization in mature TLS or the high density of TIL-PCs within tumors are indicators of good prognosis for patient survival and response to ICI treatment. Building on the well-studied B-cell dynamics in SLOs in response to infection or vaccination, the remaining gaps in our current understanding of TIL-B responses are being filled.

**Fig. 4:**
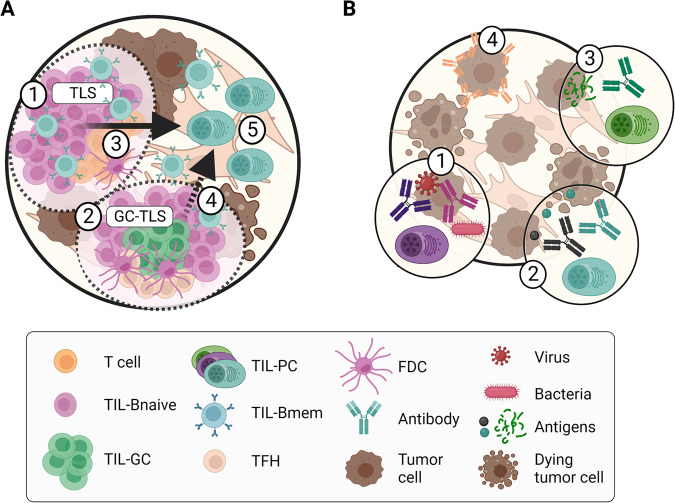
Current knowledge on TIL-B responses in tumors. **A** Different degrees of TLS maturation and organization: simple immature TLSs are composed of TIL-B and T-cell aggregates (1); GC-TLSs contain TIL-GC structures (2); reactivation of TIL-Bmem in GC-independent (3) and, to a lesser extent, GC-dependent responses (4) differentiates TIL-PCs that migrate to specific stromal areas in tumors (5). **B** TIL-B may target different types of antigens: foreign antigens present within the tumor may lead to local TIL-PC differentiation (1); self-antigens may be recognized by TIL-PC-derived antibodies (class I or class II) (2); TIL-PC-derived antibodies may recognize tumor-specific antigens or neoantigens released upon cell death (3); and some of the resulting antibodies may coat tumor cells and sensitize them to killing by NK cells or phagocytes (4). Created with BioRender.com

TIL-B organization as TLS in tumors is heterogeneous. Different degrees of TLS organization are found in tumors, with frequent immature TLS and rare GC-TLS. TIL-B dynamics and clonal diversification in tumors rely either on rare intratumoral GC-dependent reactions and more frequently on GC-independent TIL-Bmem reactivation and differentiation. In both cases, the resulting TIL-B activation relies on the assistance of T cells, and the differentiated TIL-PCs are supported by a supportive stroma, providing a survival niche for local antibody production (Fig. 4A). In tumors, several studies have reported the ability of TIL-B cells to target different types of antigens: foreign antigens, self-antigens, or tumor-specific antigens. The antigen specificity of TIL-PC-derived antibodies may be acquired through affinity maturation in a GC-dependent process or may preexist in germline precursors. Self-reactive TIL-PC-derived antibodies likely develop because of the overexpression of certain self-antigens in the TME. TIL-PC-derived antibodies may coat tumor cells, targeting those cells for antibody-dependent cell cytotoxicity or phagocytosis upon recruitment of NK cells or phagocytes, respectively (Fig. 4B).

Despite recent progress in our understanding of TIL-B responses, there are still many pending questions regarding the orchestration of B-cell responses within tumors.

### Perfect GC-TLS?

Since in most cases GC-TLS lack key GC-SLO features such as a DZ-LZ organization, we postulate that even in the rare occasions when GC-TLS are found in tumors, they do not effectively facilitate the differentiation of numerous high-affinity antigen-specific TIL-Bmem and TIL-PC. We propose that some circumstances induce the formation of “perfect” GC-TLS that adopt the spatial organization of GC-SLO to sustain affinity maturation and generation of long-lived TIL-Bmems and high-affinity TIL-PCs from de novo activated Bnaive cells (low levels of SHM) and reactivated Bmem cells (high levels of SHM).

How can perfect GC-TLS be generated? Denton et al. [76] studied ectopic GC formation in lung tissue in a murine model of respiratory viral infection. They revealed major differences in ectopic TLS formation compared to conventional GC-SLO, which may be of interest to understand what is missing for perfect GC-TLS formation in tumors. They demonstrated that upon influenza virus infection, type I IFN production induced lung fibroblasts to produce CXCL13, allowing the CXCR5-dependent recruitment of B cells, which in turn formed TLS and ectopic GCs in situ. In the absence of viral infection, the CXCR5-CXCL13 signaling axis was not sufficient to generate B-cell trafficking and GC formation. The same mechanism may occur in the TME, where CXCL13 production recruits TIL-B cells but may not be sufficient to generate strong and long-lasting GC-TLS reactions. Denton et al. revealed that only the combination of IFNβ and cGAMP was able to induce pulmonary GC formation. Those signals, and others that need to be elucidated, may be missing upon GC-TLS development in tumors.

### Are ICIs generating perfect GC-TLS?

ICI treatment most likely induces direct and indirect activation of TIL-B, but the precise mechanism and impact on TIL-B and TLS are still unclear. Are ICIs triggering the development of perfect GC-TLS?

ICI treatment induces lymphocyte recruitment into tumors in a murine melanoma model, as reported by Asrir et al. [77]. Lymphocyte accumulation might allow the development of more consistent and organized TLS. ICI treatment also triggers TIL-B clonal expansions. Helmink et al. [31] revealed that Bmem cells were the most enriched B-cell subset in responder patient samples (51%), followed by PC (16.3%), compared to nonresponder patients (23.7% and 1.7%, respectively). The proportion of clonally expanded B cells was higher for responder patients, while nonresponder patients had mostly clonotypes of small size. A high density of Bmem cells may be the consequence of GC-TLS activation and export of effector TIL-B subsets or more likely the consequence of reactivated TIL-Bmem clonal expansion and GC-independent differentiation into TIL-PCs.

Perfect GC-TLS require the presence of functional TFH cells for affinity-based selection of TIL-GC B cells. TFH cells express high levels of PD-1 [78] and are therefore targeted by anti-PD-1 ICI treatment. Could TFH activation by anti-PD-1 blocking antibodies transform GC-TLS into perfect GC-TLS? In a recent study, Herati et al. [79] studied B and T-cell responses to seasonal influenza vaccines in anti-PD-1-treated cancer patients. Anti-PD-1-treated patients displayed more circulating TFH (cTFH) cell responses than untreated patients. Additionally, patients who had experienced immune-related adverse events (irAEs) following anti-PD-1 treatment had more robust vaccine-induced cTFH responses than patients without irAEs. Thus, anti-PD-1 treatment may boost TFH cell activity in TLS. PD-1 expression by TFH cells has been shown to promote the confinement of TFH cells within the GC territory and strengthen the stringency of GC affinity selection [78]. Therefore, unleashing PD-1 with ICIs might increase TFH cell recruitment to GCs, increase TCR ligand sensitivity and diminish the stringency of B-cell affinity maturation. Are post-ICI GC-TLS functional but with a drastic decrease of selection stringency?

### What is the stability of TIL-B responses over time?

The systemic repercussions of TIL-B responses can be tracked by studying the antigen reactivity of serum antibodies in cancer patients. How stable are those systemic antibody responses? Lee et al. [80] studied serum antibody responses against influenza virus antigens across multiple years of exposure and repeated vaccinations. They reported persistent antibody lineages that accounted for 70% of the serum response over 5 years and that cross-neutralized a divergent H5N1 strain, thereby revealing that Bmem clones expressing cross-reactive BCR are recalled regularly to provide immune protection. In cancer patients, serum reactivity analysis was mostly performed at a single time point, upon cancer diagnosis. Studying the stability of cancer-specific serum reactivity across time may demonstrate the establishment of robust, long-lasting antitumor B-cell responses and may help us understand how to reinvigorate antitumor responses.

In the future, next-generation integrative B-cell immunology methods will yield a precise understanding of TIL-B organization as GC-TLS, antigen-specific clonal expansions and differentiation into TIL-PCs before and after treatment with ICIs. Such knowledge will be a remarkable asset to design innovative immunotherapy strategies specifically targeting TIL-B cells, with the potential to durably eliminate cancer.
